# Bone metastasis of a gastrointestinal stromal tumor: A report of two cases

**DOI:** 10.3892/ol.2015.2976

**Published:** 2015-02-17

**Authors:** KAYO SUZUKI, TAKETOSHI YASUDA, KAORU NAGAO, TAKESHI HORI, KENTA WATANABE, MASAHIKO KANAMORI, TOMOATSU KIMURA

**Affiliations:** 1 Department of Orthopedic Surgery, University of Toyama, Toyama, Toyama 930-0194, Japan; 2 Department of Orthopedic Surgery, Iiyama Red Cross Hospital, Iiyama, Nagano 389-2295, Japan; 3 Department of Human Science, University of Toyama, Toyama, Toyama 930-0194, Japan

**Keywords:** gastrointestinal stromal tumor, bone metastasis, surgery, imatinib

## Abstract

Gastrointestinal stromal tumors (GISTs) are the most frequently diagnosed mesenchymal tumors of the GI tract. GISTs usually arise from the stomach, followed by the small intestine, rectum and other locations in the GI tract. The most common metastatic sites are the liver and peritoneum, whereas GISTs rarely metastasize to the bone. Although a small number of previous studies have described bone metastases originating from GISTs, the true prevalence is yet to be elucidated. The present study describes two cases of bone metastasis in patients with GISTs and reviews the relevant literature. Case one was of a 78-year-old male who presented with bone metastasis to the femoral neck five years after the resection of a GIST. The metastasis was completely resected and the patient remains alive nine years after the initial diagnosis of the GIST. Case 2 was of a 41-year-old male who presented with bone metastases to the ribs following resection of GISTs seven and 17 years earlier. The metastases were completely resected and the patient remains alive 17 years after the initial diagnosis. In total, only 10 cases of GISTs with metastases to the bone have been reported in the English literature. The possibility of bone metastases originating from a GIST should be considered during clinical follow-up, particularly in the presence of liver metastases. If feasible, bone metastases should be completely surgically excised.

## Introduction

Gastrointestinal stromal tumors (GISTs) are the most frequently diagnosed mesenchymal tumor of the GI tract, accounting for 1–3% of all malignant GI tumors (1). They are defined pathologically as c-Kit-positive mesenchymal spindle cell or epithelioid neoplasms (1). GISTs usually arise from the stomach (60–70%), followed by the small intestine (20–30%), the rectum (5%) and the esophagus (<5%), with an estimated prevalence of 11–14 cases per 1,000,000 individuals (2,3). Only 70% of patients with GIST are symptomatic. While 20% are asymptomatic and the tumors are detected incidentally, the remainder of cases are detected during autopsy. The most common symptoms of GISTs are nausea, vomiting, abdominal discomfort, bleeding from the GI tract and weight loss (4). The most common metastatic sites are the liver and peritoneum, whereas GISTs are rarely found to metastasize intracranially or to the lymph nodes, lungs or subcutaneous tissue, as described in a limited number of previous case studies (5–7). Although a small number of studies have described bone metastases originating from GISTs (6,8–14), the true prevalence remains unknown (15,16). At present, the standard therapy for GIST is surgical complete resection, which is is possible in ~85% of patients. However, GIST recurrences and metastases are identified in 50% of patients following complete resection. Thus, for the treatment of these advanced or unresectable GISTs, the selective tyrosine kinase inhibitors, imatinib or sunitinib, are used (4,17). The overall survival rates for patients with advanced or unresectable GISTs who are treated with imatinib is 55% (18). The present study describes two cases of bone metastasis in patients with GISTs and reviews the relevant literature. Written informed consent was obtained from both patients.

## Case reports

### Case one

**Figure 1:**
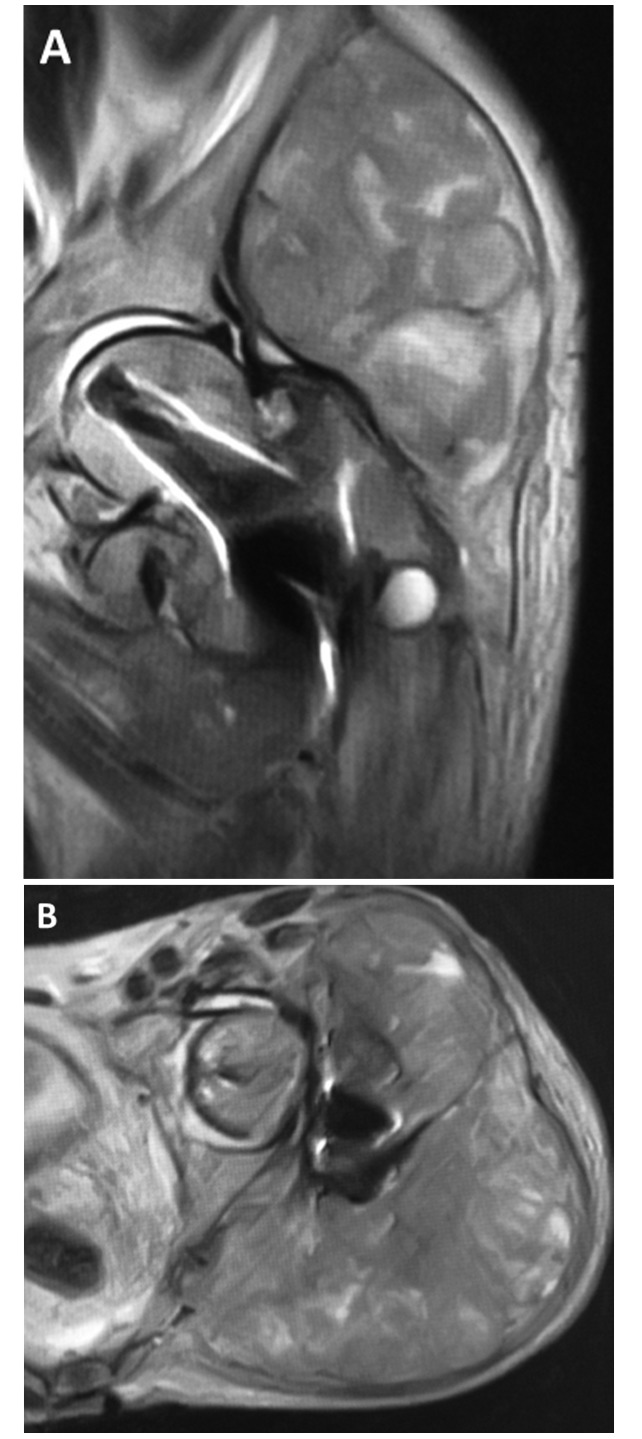
Case one: Magnetic resonance imaging of the left hip. (A) Coronal and (B) axial T2-weighted imaging of the left hip revealing a large mass extending from the femoral neck to the buttock, demonstrating heterogeneous signal hypo- and hyperintensity.

**Figure 2:**
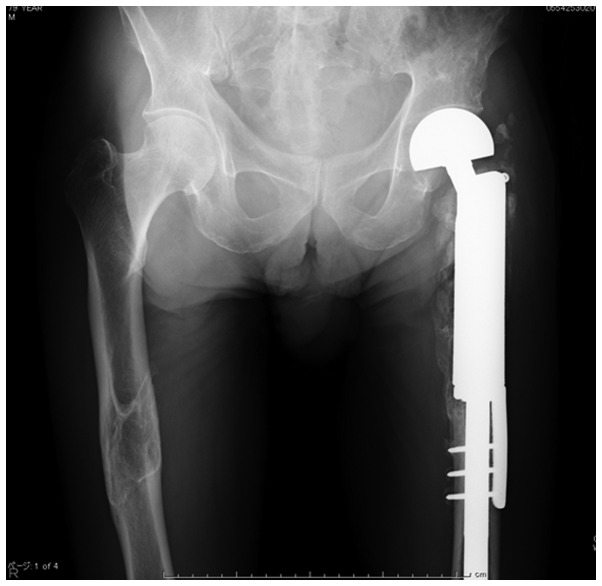
Case one: Radiographical observations following surgery. The metastatic bone lesion was resected and reconstructed using a tumor prosthesis.

**Figure 3:**
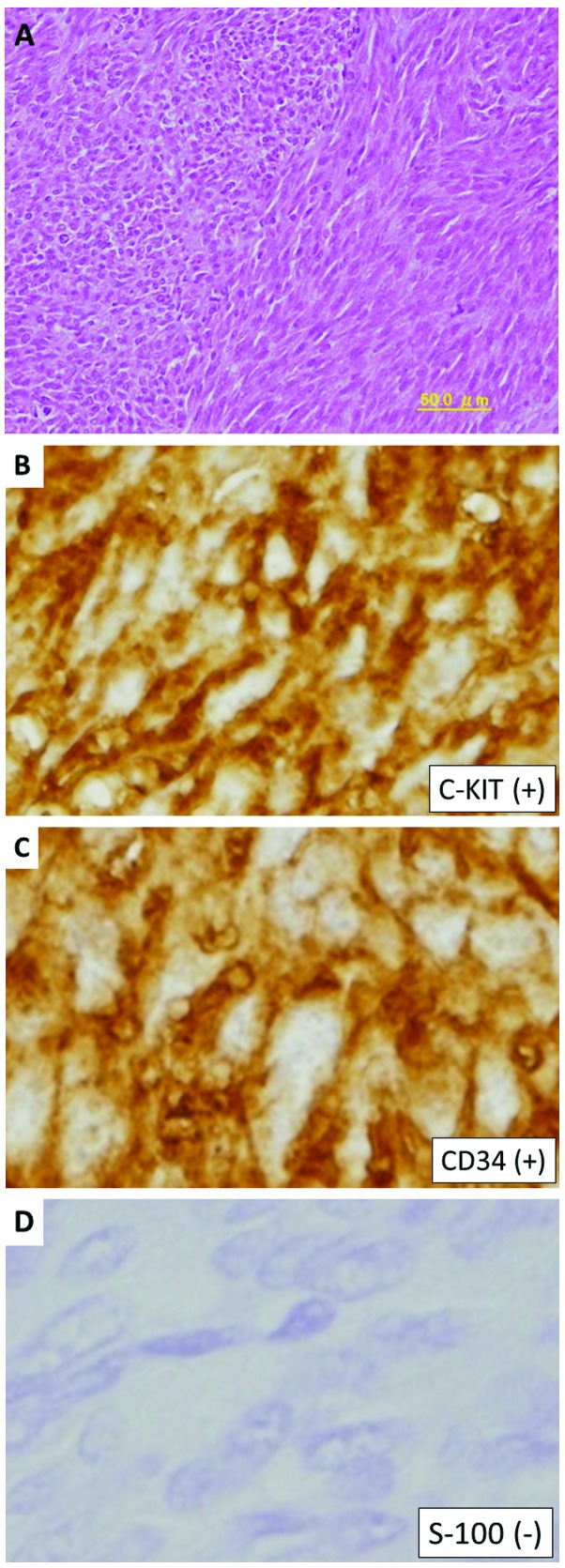
Case one: Histopathological appearance of the surgical specimen. (A) The resected mass revealing a hypercellular tumor with spindle-shaped cells. Mitotic index was 70 per 50 high-power fields (hematoxylin and eosin stain; scale bar, 50 μm). Immunohistochemical staining for (B) c-KIT and (C) cluster of differentiation 34 revealing diffuse staining of the cell membrane. (D) Negative staining results obtained for S-100.

A 78-year-old male had previously undergone surgical removal of a stomach GIST in 2004. The patient subsequently fell and fractured the left femoral neck in September 2009. Although osteosynthesis of the femoral neck was performed at a previous hospital, union of the femoral neck was not achieved and the patient reported a painful, swollen mass in the left buttock. Plain radiographs revealed non-union at the surgical site and a mass in the soft-tissue of the buttock. Magnetic resonance imaging of the hip identified a giant mass spreading around the left buttock from the femoral neck (Fig. 1). A biopsy was performed at the previous hospital, and a diagnosis of synovial sarcoma was suggested. The patient was admitted to Toyama University Hospital (Toyama, Japan) in 2009, where wide resection of the tumor and reconstruction of the hip joint using a tumor prosthesis were performed (Fig. 2). Histopathological examination revealed a hypercellular spindle-cell neoplasm, with a severe mitotic index of 70 per 50 high-power fields (Fig. 3A). Immunohistochemically, the tumor cells were positive for c-KIT and cluster of differentiation (CD)34, but negative for S-100 protein (Fig. 3B–D). Chemotherapy was administered, which consisted of treatment with imatinib (300 mg, daily) for four years. The patient remains alive at four years post-surgery and is able to walk unaided.

### Case two

**Figure 4:**
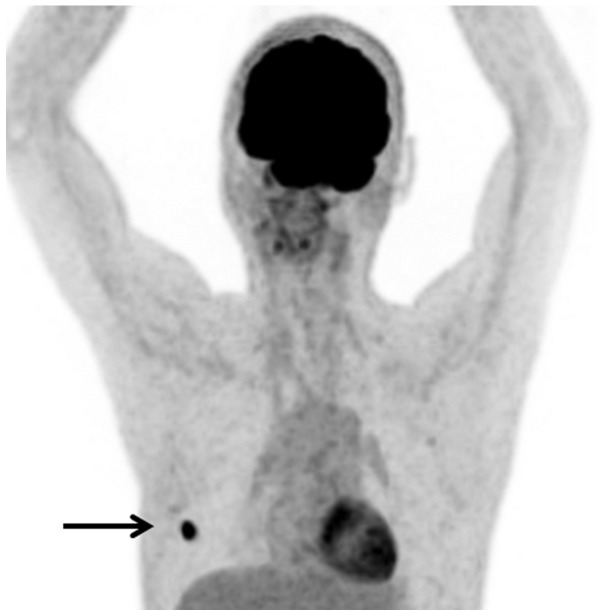
Case two: Positron emission tomography-computed tomography revealing rib metastasis (arrow).

A 41-year-old male had previously undergone surgical removal of a GIST of the rectum at Nagoya University Hospital (Nagoya, Japan) in 1997. A local recurrence was identified and resected in 2004. Positron emission tomography (PET)-computed tomography (CT) revealed a metastatic bone lesion in the left fourth rib. The bony lesion was completely resected in April 2007 at Toyama University Hospital and the patient was treated with imatinib (400 mg, daily) for three years. A stable disease state was subsequently maintained with the administration of imatinib (400 mg, daily) for five years. However, metastatic renal and liver lesions were identified and completely resected at Fukui-ken Saiseikai Hospital (Fukui, Japan) in 2012. Following resection, the patient was administered imantinib (400 mg, daily). In 2013, PET-CT revealed the presence of metastasis in the right fifth rib (Fig. 4), which was subsequently completely resected at Toyama University Hospital in December 2013. Histopathological examination revealed oval-shaped spindle cells with fascicular proliferation. Immunohistochemical analysis identified that the tumor cells were positive for c-Kit and CD34. The patient remains alive 17 years after the initial diagnosis.

## Discussion

GISTs are pleomorphic mesenchymal tumors of the GI tract, consisting of spindle cells, epithelioid cells, or a combination of these cells, expressing c-KIT protein and, in the majority of cases, CD34 (4,19). The gold standard of therapy for GISTs is surgical resection of the local disease. The aim of treatment is complete resection of the lesion whilst avoiding tumor rupture. Survival rates are determined by tumor size, and not the presence of negative microscopic surgical margins. Complete surgical resection is achieved in ~85% of patients, however, 50% go on to develop recurrence or metastasis following the surgery (17). The five-year survival rate for GIST patients is ~50%, while the median time to recurrence following resection of a primary high-risk GIST is two years (4). However, since the introduction of imatinib as a molecular-targeted therapy, marked improvements have been made in the rates of progression-free and overall survival. Despite this, GISTs have a high-risk of metastatic relapse.

The most common metastatic sites are the liver (65%) and peritoneal surface (50%) (17), while GISTs rarely metastasizes to the bone. In a series of studies concerning metastatic GISTs, the incidence of bone metastasis was ~3% (15,16,20). However, the specific characteristics of patients with bone metastasis have not yet been identified. In total, only 12 cases of GISTs with metastasis to the bone, including those in the present study, have been reported in the English literature (Table I) (6,8–14). In the 12 literature cases, the most common site of bone metastasis was the spine (6/12; 50%). Six cases that presented with metastases to the spine did not undergo resection of the lesions. This suggests that metastatic spinal lesions are typically unresectable due to the infiltration of surrounding nervous tissue. As a result, four of the six patients succumbed to the disease within 17–90 months (mean, 51.5 months). In total, 11 of the 12 cases (92%), excluding case 11 (case one of the present study), exhibited liver metastases at the time that the bone metastases were identified. Therefore, in the event that liver metastasis is identified during clinical follow-up, an examination for the presence of bone metastases should also be performed. The time between initial diagnosis and the development of bone metastasis varies. The location of the primary site also varies. In the present study, evidence of bone metastasis originating from a GIST was identified five and nine years after the diagnoses of primary stomach and rectal GISTs, respectively.

A diagnosis of bone metastases originating from a GIST is usually based upon clinical findings, bone fractures or pain. An early diagnosis of bone metastases is desirable in order to avoid a marked reduction in quality of life. PET-CT is useful for the early diagnosis of bone metastases that originate from GISTs. Upon PET-CT, metastatic bone lesions exhibit high ^18^F-fluorodeoxyglucose avidity, similar to that of the liver and other metastatic sites (16). In the present study, PET-CT proved useful in achieving a diagnosis.

Imatinib has revolutionized the treatment of advanced GISTs (17), enabling the long-term survival of patients. However, the optimal treatment for bone metastases originating from GIST is yet to be elucidated. Of the 12 patients with GIST-induced bone metastases described to date, four were treated with imatinib alone, four received radiotherapy and four underwent surgical resection of the lesion (Table I). The choice of therapy alters according to a number of factors. In the event that multiple lesions develop despite the use of imatinib, another tyrosine kinase inhibitor (TKI) may be administered. Treatment options for patients with progressive disease or widespread systemic disease and a good performance status include continuation of imatinib at the same dose, dose escalation to 800 mg/day in the absence of severe adverse drug reactions, or switching to sunitinib, a multi-targeted receptor TKI (4). In addition to TKIs, radiotherapy can be used in patients with bone metastases for palliative purposes (12). Bisphosphonates are also effective for the management of bone metastases (11). If the metastatic lesion is isolated and occurs in a resectable site, imatinib may prove useful. In cases where the tumor remains resectable, imatinib can be continued indefinitely until there is evidence of tumor progression following resection. In fact, long-term survival was achieved in the present study by resecting an isolated bone lesion.

In conclusion, although bone metastases originating from GISTs are rare, the likelihood of identifying metastases in unusual sites is increasing due to the prolonged survival of patients with the tumors and the introduction of imatinib therapy. The risk of bone metastases from GISTs should be considered during long-term follow-up, particularly in the presence of liver metastases. Furthermore, since side-effects from TKIs are common, bone metastases should be completely surgically excised if possible.

## Figures and Tables

**Table I:** Clinical characteristics of patients with bone metastases from a gastrointestinal stromal tumor.

Case no. (ref.)	Age, years/gender	Primary site	Site of metastases	Period of bone metastases^a^, months	Site of bone metastases	Therapy for bone metastases	Outcome^a^, months
1 (5)	57/M	Rectum	Liver, bone	At initial diagnosis	Spine	TKI	DOD, 17
2 (7)	58/M	Small intestine	Liver, bone	28	Clavicle, spine	TKI, radiation	AWD, 47
3 (8)	54/M	Rectum	Liver	24	Scapula	TKI, resection	AWD, 120
4 (9)	57/M	Small intestine	Liver	49	Humerus	TKI, resection, radiation	DOD, 55
5 (10)	62/M	Small intestine	Liver, bone	At initial diagnosis	Spine, pelvis, rib	TKI, radiation, zoledronic acid	DOD, 33
6 (10)	82/F	Stomach	Liver, bone	At initial diagnosis	Spine, pelvis	TKI	AWD, 48
7 (10)	54/F	Small intestine	Liver	84	Spine, pelvis, rib	TKI, zoledronic acid	DOD, 96
8 (11)	83/M	Rectum	Liver, bone	12	Femur	TKI, radiation, zoledronic acid	AWD, NA
9 (12)	53/M	Esophagus	Lung, bone	At initial diagnosis	Humerus	TKI	AWD, 2
10 (13)	69/F	Stomach	Liver, bone	12	Spine	TKI	DOD, 60
11 (Present study)	78/M	Stomach	Bone	48	Femur	TKI, resection	NED, 72
12 (Present study)	41/M	Rectum	Liver, bone, kidney	108	Rib	TKI, resection	NED, 204

a Period from initial diagnosis.

M, male; F, female; TKI, tyrosine kinase inhibitor; AWD, alive with disease; DOD, died of disease; NED, no evidence of disease; NA, not available.
